# Secretory Production of Mature Protein-Glutaminase from *Bacteroides helcogenes* in *Bacillus subtilis*

**DOI:** 10.1007/s12010-026-05637-6

**Published:** 2026-03-02

**Authors:** Gudrun Horstmann, Nicole Roth, Eva Pross, Jana Senger, Ines Seitl, Lucas Kettner, Lutz Fischer

**Affiliations:** Department of Biotechnology and Enzyme Science, University of Hohenheim, Institute of Food Science and Biotechnology, Garbenstrasse 25, 70599 Stuttgart, Germany

**Keywords:** Protein-glutamine glutaminase, Bacillus subtilis, Protein deamidation, Recombinant expression, Propeptide hydrolysis

## Abstract

The recombinant production of the protein-glutaminase (PG) from *Bacteroides helcogenes* (PGB) was investigated in the three *Bacillus subtilis* strains: 168, RIK1285 and the isolated strain 007. *B. subtilis* 007 produced the highest PG activity (7.6 ± 0.7 µkat L_Culture supernatant_^−1^) in shake flask cultivations and, thus, was used for further investigations. As a wild-type strain, *B. subtilis* 007 exhibited high extracellular proteolytic activity and formed large amounts of foam in a bioreactor cultivation. While the proteolytic activity would be favorable for the extracellular cleavage of PGB’s propeptide, foaming is undesirable and should be prevented. Therefore, the influence of the two foam-demolishing substances anti-foaming agent 204 and rapeseed oil on the PGB production was investigated in bioreactor cultivations. A PGB activity of 11.8 ± 1.1 µkat L_Culture supernatant_^−1^ was obtained using anti-foaming agent 204, while rapeseed oil led to a lower maximal PGB activity of 7.6 ± 0.2 µkat L_Culture supernatant_^−1^. PGB was partially purified from the culture supernatant by fractionated ammonium sulphate precipitation followed by hydrophobic interaction chromatography. A final yield of 14.8% was obtained, while the specific PGB activity increased from 6.7 to 28.6 nkat mg^−1^. Alternatively, PGB was purified from culture supernatant by cross-flow filtration and subsequent heating step (1 h, 60 °C) to inactivate the extracellular peptidases. Thereby, the total proteolytic activity was decreased to below 4% while the specific PGB activity increased to 32.1 ± 2.0 nkat mg^−1^. The PGB preparation obtained was applied in gluten deamidation experiments which showed that PGB deamidated a 1% (w/v) gluten suspension by 95 ± 2%.

**Supplementary Information:**

The online version contains supplementary material available at 10.1007/s12010-026-05637-6.

## Introduction

Plant-based proteins are increasingly becoming the focus of the food industry for reasons of sustainability and vegetarian diets [1]. Although the demand for plant proteins is increasing, they come with severe challenges which the food industry is trying to overcome. One of the most critical drawbacks is the poor solubility of plant proteins at a neutral pH [2]. A protein’s solubility can be improved by chemical or enzymatic deamidation of the protein’s asparagine or glutamine residues [3–5]. In this reaction, the amide group is converted to a carboxyl group. This introduction of negatively charged amino acid residues changes the protein’s surface charge and thereby can alter its technofunctional properties [6]. While initial enzymatic deamidation studies focused on peptidoglutaminases (EC 3.5.1.43; [5]) and transglutaminases (EC 2.3.2.13; [7]), both enzymes were found unsuitable due to limited protein deamidation or unwanted side activities. A new enzyme was found in *Chryseobacterium proteolyticum* in 2000, which was able to deamidate glutamine residues in proteins and was, therefore, named protein-glutaminase (PG, EC 3.5.1.44, [8]). The PG was considered to have a great potential for the food industry. Several studies investigated protein deamidation using PG from *C.* *proteolyticum* (PGC), and its positive influence on the technofunctional properties of different proteins originating from oats [9], soy [10] or gluten [4] became apparent. The solubility of hardly soluble proteins was especially improved. As a result of the increased solubility, foaming and emulsion behavior was also enhanced, as soluble proteins diffuse to water–air or water–oil interfaces and form a stabilizing network there [9, 11]. PGC is produced as an inactive pro-protein and targeted for secretion by its signal peptide. After pro-PGC is secreted, the propeptide is cleaved by extracellular peptidases, resulting in the active PGC [8]. So far, PGC is the only commercially available PG.

The discovery of new PGs is of great interest to scientists and industry professionals looking to expand the range of applications. A novel PG, PGB, was found in *Bacteroides helcogenes* in an *in-silico* screening using the Basic Local Alignment Tool [12], based on the PGC gene sequence. PGB shares 47% sequence homology with PGC [13]. The former was cloned and expressed in its proform (pro-PGB) in *E. coli* and was found to be active even without cleavage of the propeptide.

Horstmann et al. [13] compared mature PGC and pro-PGB with regards to solubilizing wheat gluten, an extensively studied model protein that is insoluble in aqueous systems. Pro-PGB deamidates wheat gluten faster and to a higher degree than mature PGC. Additionally, pro-PGB is more stable at elevated temperatures and exhibits maximal activity at a slightly acidic pH of 5.5 [13]. In vitro cleavage of the propeptide increased its deamidating activity 17-fold [14].

The first attempts at homologous expression were conducted using *C. proteolyticum* and showed low production yields [15]. Consequently, recent research has focused on developing improved production processes using different hosts, such as *C. glutamicum* [16], *E. coli* [17], and *B. subtilis* [18]. *B. subtilis* is a well-known organism with qualified presumption of safety (QPS) status. It is used for secretory production of numerous proteins for applications in the food industry [19, 20].

In *B. subtilis*, the expression level of a target protein can be improved by varying the start codon, which influences mRNA stability [21]. While translation of most *B. subtilis* proteins (75–86%) is initiated by AUG [22], the remaining proteins are initiated by alternative start codons [21–23]. In our study, the start codons AUG and GUG were investigated in *B. subtilis* strains with different extracellular proteolytic activity, and the achieved deamidation activity was compared.

Using *B. subtilis* as a production host achieves propeptide cleavage during production, as demonstrated for pro-PGC [18, 24] using *B. subtilis* 168. This strain secretes seven peptidases that can cleave the propeptide of PGs. Although different *Bacillus* strains share the same set of peptidase genes, their extracellular proteolytic activity can differ. For this study, *Bacillus subtilis* 007, a wild-type strain reported recently to exhibit high extracellular proteolytic activity [25], was used for PGB production. However, wild-type strains often exhibit undesirable behavior, such as excessive foaming during bioreactor cultivation. This may be due to the secretion of surface-active compounds, such as surfactin, as has been reported for *Bacillus subtilis* [21, 22] Excessive foaming can be mitigated by adding foam-deconstructing compounds during cultivation. In our study, we compared the effect of a commercial anti-foaming preparation to that of regular rapeseed oil, a topic rarely addressed in the literature.

Because producing and applying mature PGB became interesting due to previous gluten deamidation experiments, its production in the wild-type strain *Bacillus subtilis* 007 was investigated. Researchers have not reported recombinant secretory production of a PG in a *B. subtilis* wild-type strain before.

## Materials and Methods

### Construction of Expression Plasmids for Recombinant PGB Production

The following constructs and strains were used for expression experiments (Table 1).

**Table 1:** Genes, constructs and organisms used for the PGB production

Construct name	Promoter	Start codon	Signal sequence
pLF_GUG_pro-PGB	AprE	GUG (Valine)	PhoD
pLF_AUG_pro-PGB	AprE	AUG (Methionine)	PhoD
Expression host	Genotype		Source/Manufacturer
* B. subtilis* RIK1285	*trpC2 lys1 aprEΔ3 nprR2 nprE18*		Takara BioInc, Japan
* B. subtilis* 168	*trpC2*		DSM 23778
* B. subtilis* 007	Isolate		DSM 118688 [25]

The genomic DNA of *Bacteroides helcogenes* was purchased from the German Collection of Microorganisms and Cell Cultures (DSM 20613, Braunschweig, Germany). The native pro-PGB gene (GenBank Accession: CP002352.1; Protein ID: ADV44662.1; UniProt No: E6SWX4_BACT6) was amplified from the genomic DNA by polymerase chain reaction (PCR) using the ExTaq polymerase (Takara BioInc, Germany; primers: Pro-PGB_*BssH*II_fw and PGB_*Xho*I_rv, Table S1). The restriction digestion of the PCR product was carried out with *BssH*II and *Xho*I (New England Biolabs, Germany). The ligation into the pLF vector (digested with *BssH*II and *Xho*I) was done using the T4-DNA ligase (Thermo Fisher Scientific, USA). The pLF-vector is an *E. coli/B. subtilis* shuttle vector containing ampicillin and neomycin resistance genes for selection in *E. coli* and *B. subtilis*, respectively. The constitutive P_aprE_ promoter and the PhoD signal sequence were used for gene expression [26], both originating from *B. subtilis*. The pLF expression vector initially contained GUG as a start codon upstream of the PhoD signal peptide. This codon was exchanged to AUG via site-directed mutagenesis [27] using Q5 polymerase (New England Biolabs, Germany, primers: QC_M1V_fw and QC_M1V_rv; Table S1). *E. coli* XL-1 Blue cells were transformed with both plasmids (holding GUG or AUG start codons, respectively) by heat-shock transformation. The plasmids were isolated from single colonies using the GeneJET Plasmid-Miniprep Kit (Thermo Fisher Scientific, Hampton, USA). Correct cloning was verified by sequencing (Eurofins Genomics, Germany).

### Transformation of *B. subtilis*

Prior to each cultivation, *B. subtilis* strains were freshly streaked onto lysogeny broth agar plates (10 g L^−1^ Tryptone, 5 g L^−1^ yeast extract, 5 g L^−1^ sodium chloride, 15 g L^−1^ agar) and grown at 37 °C for 18 h. Plasmids were prepared for transformation via rolling circle amplification (TempliPhi 100 Kit, GE Healthcare Life Sciences, Germany) and 1 µg of amplified plasmid DNA was used for transformation. The latter was done using the overnight cultures prepared and the amplified plasmid, according to Vojcic et al*.* [28] but without the addition of histidine as this was not necessary for the strains used in this study. After transformation, cells were plated onto lysogeny broth agar plates containing neomycin (7.5 µg mL^−1^) for selection.

### Shake Flask Cultivation of *B. subtilis* for Secretory PGB Production

The *B. subtilis* strains 168 (DSM 23778), RIK1285 [29] and 007 [25] were investigated for secretory PGB production. Cultivation experiments were conducted in complex media, as described by Zhang et al*.* [30]. Precultures of 5 mL were grown in glass tubes using seed medium (40 g L^−1^ sucrose, 30 g L^−1^ soy peptone, 6 g L^−1^ KH_2_PO_4_, 2 g L^−1^ MgCl_2_ * 6 H_2_O, 7.5 µg mL^−1^ neomycin for strains containing plasmid) at 37 °C for 20 h. The main cultures were inoculated to an optical density (OD_600_) of 0.1 in 50 mL fermentation medium (70 g L^−1^ sucrose, 50 g L^−1^ soy peptone, 5 g L^−1^ KH_2_PO_4_, 3 g L^−1^ MgCl_2_ * 6 H_2_O and 7.5 µg mL^−1^ neomycin for strains containing plasmid) and incubated on an orbital shaker (110 rpm) at 30 °C for 5 days. The respective strains devoid of the expression plasmid were cultivated as negative controls without neomycin. Samples were taken regularly, and the cell growth was tracked by OD_600_ measurement. Cultivation samples were centrifuged (12.857 × *g*, 4 °C, 5 min) and the supernatant was desalted via PD-10 columns (VWR International GmbH, Germany) into 100 mM 2-(*N*-morpholino)ethanesulfonic acid (MES) buffer (pH 5.5, Carl Roth GmbH & Co. KG, Germany) for the determination of PG activity. The desalted cell-free supernatant was stored at −20 °C and investigated by sodium dodecyl sulfate polyacrylamide gel electrophoresis (SDS-PAGE) analysis.

When the necessity of an anti-foaming agent became obvious, anti-foaming agent 204 (Merck KgaA, Germany) was tested for its influence on growth and PG activity at shake flask scale. Cultivations were conducted as described previously, and stopped after 68 h. An initial concentration of 0.01% (v/v) anti-foaming agent 204 was added. In the first experiment, the concentration of anti-foaming agent 204 was increased in two steps to 0.3 and 0.7% (v/v) after 44 and 52 h, respectively. In the second experiment, the anti-foaming agent 204 concentration was increased over time from 0.01 to 0.2% (v/v) within 68 h. *B. subtilis* 007 was cultivated without any addition of anti-foaming agent as a control.

### Foam Control During the Bioreactor Cultivation of *B. subtilis* 007 for Secretory PGB Production

The *B. subtilis* 007 strain was used for secretory PGB production in stirred-tank bioreactors in duplicate with a working volume of 0.8 L (Multifors, Infors, Switzerland). Two consecutive precultures of 10 and 100 mL were carried out in seed medium at 37 °C for 10 and 14 h, respectively. In order to investigate the effect of different anti-foaming agents in bioreactor cultivations, the second pre-culture already contained 0.01% (v/v) of either anti-foaming agent 204 (Merck KgaA, Germany) or rapeseed oil (Rapso® Rapso Österreich GmbH, Austria). The cells were grown in the bioreactor cultivation in fermentation medium with neomycin (7.5 µg mL^−1^) and anti-foaming agent 204 (0.01% (v/v)) or rapeseed oil (0.25% (v/v)). The pO_2_ was regulated to > 30% by adjusting the stirrer speed and air flow with a starting aeration rate of 0.8 vvm. A pH of 6.9 ± 0.1 was maintained with 2 M H_3_PO_4_ and 2 M NaOH. Samples were taken regularly, centrifuged (7912 × g, 10 min, 4 °C) and the pellet and supernatant were stored separately at −20 °C. Pelleted cells were dried for 3 h at 70 °C and 1 mbar for the determination of the bio dry mass. Samples containing rapeseed oil were dried for 6–8 h at 70 °C and 1 mbar. Sample preparation for PG and proteolytic activity determinations are described in the respective sections.

The excessive foaming in the reactors was controlled by the manual addition of the respective anti-foaming agent. Anti-foam agent 204 and rapeseed oil were added manually in the respective cultivation whenever needed. A peristaltic pump was installed for the automated addition of rapeseed oil overnight, and rapeseed oil was added every 5 min for 10 s with a flow of 47 µL s^−1^ from 11 to 14 h and every 30 min from 14 to 25 h and 36 to 47 h. The automated dosage of anti-foam agent 204 overnight was not necessary. The cultivations were stopped after 53 h.

### Ammonium Sulphate Precipitation and Hydrophobic Interaction Chromatography of PGB

A fractionated ammonium sulphate precipitation of proteins in *B. subtilis* 007 culture supernatant (shake flask cultivation; final sample) was conducted, as described by Horstmann et al*.* [13]. Using a 4 M ammonium sulphate stock solution, the culture supernatant was first brought to an ammonium sulphate concentration of 0.8 M and stirred on ice for 60 min. After centrifugation (12.857 × g, 4 °C, 10 min), the ammonium sulphate concentration in the supernatant was increased to 2.2 M and stirred again on ice for 60 min. After centrifugation, the pellet was collected and dissolved in 7 mL binding buffer (25 mM sodium phosphate, pH 7 containing 1.3 M ammonium sulphate) and filtered (0.45 µm) prior to hydrophobic interaction chromatography (HIC). The sample (5 mL) was loaded onto a 25 mL HIC column (Toyopearl Phenyl-650 M, Tosoh Bioscience GmbH, Germany). Weakly interacting proteins were washed from the column with binding buffer for 3.2 column volumes. Proteins were eluted from the column by decreasing the ammonium sulphate concentration in a linear gradient over 8 columns volumes (elution buffer: 25 mM sodium phosphate buffer; pH 7). An additional elution step with 100% elution buffer (3 columns volumes) was done to elute strongly interacting proteins. A flow rate of 3 mL min^−1^ was used for the whole procedure. Fractions were desalted with PD-10 columns into 100 mM MES buffer (pH 5.5) prior to the PG activity determination and SDS-PAGE analysis.

### Cross-flow Filtration and Heat Inactivation of Peptidases

The culture supernatants from the *B. subtilis* 007 bioreactor cultivations containing PGB were concentrated using a cross-flow filtration cassette (VivaFlow, Sartorius, Germany) with a 30 kDa molecular weight cut-off (MWCO). The cassettes were connected to a peristaltic pump and filtration was implemented at 4 °C. In the case of the supernatant from the bioreactor cultivation treated with rapeseed oil as an antifoaming agent, a 100 kDa MWCO filtration step was taken before the concentration step at 30 kDa to remove any turbidities from the supernatant. After the samples had been concentrated, it was heated to 60 °C for 1 h to inactivate peptidases. This procedure was evaluated by determining the residual proteolytic activity, PG activity and by SDS-PAGE analysis.

### Deamidation of Gluten Using PGB

The filtered and concentrated bioreactor culture supernatant containing anti-foaming agent 204 was used for the deamidation of gluten (Nestlé Product Technology Centre, Singen, Germany). Suspensions of 2.5 (w/v) and 1% (w/v) gluten protein were prepared in 100 mM MES (pH 5.5). Deamidation experiments were done at a 500 µL scale at 50 °C, 1000 rpm for 3 h in a thermoshaker using different PGB activities of 0.06–0.9 nkat mL^−1^. The respective gluten suspensions without added PGB were equally incubated as references. The reaction was stopped by incubation at 95 °C for 5 min. After centrifugation (12.857 × g, 5 min, 4 °C), the ammonia released was determined using the Ammonia (Rapid) Kit (Megazyme, Ireland), as described by Horstmann et al*.* [13]. In order to determine the maximal degree of deamidation (%DD), a 2.5% (w/v) gluten suspension was prepared and chemically deamidated in 2 M HCl at 95 °C for 3 h. The %DD was calculated with Eq. 1.

1 $$\mathrm{Degree}\;\mathrm{of}\;\mathrm{deamidation}\;\lbrack\%\mathrm{DD}\rbrack\;=\;\frac{\mathrm{Enzymatically}\;\mathrm{released}\;\mathrm{ammonia}\;\lbrack\mathrm{mM}\rbrack}{\mathrm{Maximal}\;\mathrm{releasable}\;\mathrm{ammonia}\;\lbrack\mathrm{mM}\rbrack}\;\ast\;100\%$$

### Standard Activity Assay for the Determination of PG Activity

PG activity was determined using the synthetic dipeptide Z-glutamine-glycine (Z-Gln-Gly-OH), as described by Horstmann et al*.* [13]. Samples were desalted into 100 mM MES buffer (pH 5.5) utilizing PD-10 columns. The assay was conducted in a total volume of 200 µL 100 mM MES buffer (pH 5.5) at 50 °C and at a final Z-Gln-Gly-OH concentration of 20 mM. The reaction was stopped using 20 µL of a 100 mM mercury chloride solution containing 5 mM of hippuric acid. The latter served as the internal standard for the following analysis via reversed-phase high performance liquid chromatography (Platin Blue, Knauer, Germany). The reaction mixture (5 µL) was separated on a C-18 RP column (Eurosil Bioselect 300–3 C-18, Knauer, Germany) at starting conditions of 90% H_2_O_dd_ (eluent A) and 10% acetonitrile (eluent B), containing 0.1% (v/v) trifluoroacetic acid, respectively. The following gradient elution profile was applied for elution: 0–2 min: 90% eluent A/10% eluent B; 2–9 min: 90–65% eluent A/10–35% eluent B; 9–11 min: 65% eluent A/35% eluent B; 11–12 min: 65–40% eluent A/35–60% eluent B; 12–14 min: 40% eluent A/60% eluent B; 14–15 min: 40–90% eluent A/60–10% eluent B; and 15–18 min: 90% eluent A/10% eluent B. A calibration curve was prepared for the quantification of PG activity using the product peptide Z-Gln-Gly-OH in concentrations of 0.03–2.1 mM. One katal (kat) of PG activity was defined as the production of 1 mol of Z-Gln-Gly-OH per second at the respective conditions. The occurrence of the volumetric PG activity in the cell-free supernatant of a culture is indicated by the subscript “culture supernatant” included in the respective volumetric enzyme activity.

### Determination of Proteolytic Activity Using Azocasein

The determination of proteolytic activity in the bioreactor cultivation supernatant containing anti-foaming agent 204 was done with azocasein (Megazyme International Ireland, Wicklow, Ireland). Firstly, the cultivation supernatant was desalted into 25 mM sodium phosphate buffer (pH 7). The azocasein solution (250 µL; 2.5 g L^−1^ in 25 mM sodium phosphate buffer (pH 7) was preincubated at 30 °C, before the addition of 50 µL of desalted and appropriately diluted supernatant. The reaction was incubated at 30 °C for 10 min and stopped by the addition of 25 µL 2 M trichloroacetic acid. After centrifugation (12.857 × *g,* 5 min, 4 °C), 190 µL of the reaction’s supernatant was transferred into a microtiter plate and mixed with 60 µL of 1 M NaOH for pH adjustment. The absorbance was determined at 450 nm. The volumetric proteolytic activity was expressed as the absorbance change (ΔA) per hour and mL of culture supernatant. The occurrence of the volumetric PG activity in the cell-free supernatant of a culture is indicated by the subscript “culture supernatant” included in the respective volumetric enzyme activity.

### Determination of Proteolytic Activity Using a Chromogenic *para*-nitroanilide (*p*NA) Substrate

The chromogenic peptide Suc-Ala-Ala-Pro-Phe-*p*NA was used for the determination of proteolytic activity in the bioreactor cultivation supernatant containing rapeseed oil. The supernatant was firstly desalted into 25 mM sodium phosphate buffer (pH 7). The assay was conducted in a total volume of 250 µL in 25 mM sodium phosphate buffer (pH 7) at 30 °C and a substrate concentration of 1.6 mM. The reaction was stopped using 25 µL of 80% (v/v) acetic acid. After centrifugation (12.857 × *g*, 5 min, 4 °C), the supernatant was diluted 1:2 in 100% ethanol to avoid disturbance by the rapeseed oil. The absorbance was measured at 340 nm. A calibration curve was prepared using *p*NA in concentrations of 0.01–0.7 mM for the quantification of the proteolytic activity. One katal of proteolytic activity was defined as the release of 1 mol of *p*NA per second. The occurrence of the volumetric PG activity in the cell-free supernatant of a culture is indicated by the subscript “culture supernatant” included in the respective volumetric enzyme activity.

### Sodium Dodecyl Sulfate Polyacrylamide Gel Electrophoresis

Samples from cultivation and purification experiments were qualitatively analyzed by SDS-PAGE using 12.5% acrylamide gels. The protein concentration of the samples was determined by Bradford assay [31] using bovine serum albumin as a reference protein. Samples containing rapeseed oil were treated using 20% (v/v) trichloroacetic acid to separate proteins from the oil. The samples were incubated at 4 °C overnight and the proteins precipitated were washed with ice-cold acetone. After resolubilizing the protein pellets in water, samples were denatured at 95 °C for 5 min in Laemmli buffer under reducing conditions before being applied onto the SDS gel. The molecular weight of proteins was estimated using the Precision Plus Protein all blue 10–250 kDa marker (Bio-Rad, Germany). Gels were stained using Coomassie brilliant blue R-250, according to Fairbanks et al*.* [32].

### Mass Spectrometry Analysis

Proteins from SDS-PAGE were further analyzed by nano liquid chromatography tandem mass spectrometry by the Core Facility Hohenheim module mass spectrometry (University of Hohenheim, Germany), as described by Horstmann et al*.* [13]. The Mascot results were transferred to Scaffold^TM^nullSoftware 4.10.0 (Proteome Software, USA).

## Results and Discussion

### Secretory PGB Production in *B. subtilis* and the Influence of the Start Codon on the PG Activity Yield

*Bacillus subtilis* was chosen for the secretory production of PGB because it enables the simultaneous hydrolysis of the propeptide by its endogenously secreted peptidases [18]. Three different *B. subtilis* strains (Table 1) were tested for secretory PGB production: the commonly used lab strain *B. subtilis* 168, the genetically modified *B. subtilis* RIK1285 [29] and the isolated *B. subtilis* 007 [25]. The strains 168 and 007 contain the complete set of genes for the eight extracellular peptidases of *B. subtilis* (namely, AprE, NprE, Bpr, Epr, Vpr, Mpr, NprB and HtrA) as well as the cell-wall-associated peptidase WprA [33]. Even though they share the same peptidases, *B. subtilis* 007 shows a higher extracellular proteolytic activity. *B. subtilis* RIK1285 is deficient in the two most abundant peptidases: AprE and NprE [29], resulting in a lower extracellular proteolytic activity compared to the other strains.

**Fig. 1:**
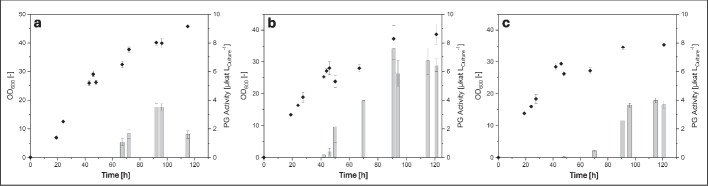
Cultivation progress of recombinant B. subtilis 168 (a), 007 (b) and RIK1285 (c) for the production of pro-PGB (expression plasmid: pLF_AUG_pro-PGB)

Protein-glutaminase activity was found in the supernatant of shake flasks cultivations of the recombinant *B. subtilis* strains 168, 007 and RIK1285, and the expected protein band for mature PG (21 kDa) was visible in the SDS-PAGE analysis for *B. subtilis* 168 and 007 (Fig. 1, S1 and S2). No PG activity or protein bands were found for the respective control strains containing no plasmid. The effect on the enzyme yield by alternating expression initiation with the start codons AUG and GUG was compared in shake flask cultivations. The majority of *B. subtilis* proteins (75–86%) are initiated by the start codon AUG [22]. Alternative start codons, such as GUG, initiate the translation of the residual proteins [21–23]. The mRNA stability and efficiency of translation can vary with the variation of the start codon [21] and according to Rocha et al*.* [22], the usage of AUG as a start codon leads to more stable mRNA and therefore to increased expression of a target protein. The initiation of the pro-PGB expression by the AUG start codon here resulted in a higher PG activity than that with GUG (Table 2).

**Table 2:** Maximal PG activities in cultivation supernatants of recombinant *B. subtilis* depending on the strain and start codon (GUG or AUG) used. Cultivations were done in shake flasks at 30 °C for 5 days

Strain	Maximal PG activity [*µ*kat L_Culture supernatant_^−1^] start codon GUG	Maximal PG activity [*µ*kat L_Culture supernatant_^−1^] start codon AUG
*B. subtilis* 168	2.3 ± 0.1	3.5 ± 0.3
*B. subtilis* 007	4.4 ± 0.3	7.6 ± 0.7
*B. subtilis* RIK1285	1.1 ± 0.2	3.9 ± 0.2

PG activity was first determined in the early stationary growth phase and increased during the same. The *B. subtilis* P_aprE_ promoter, which is used for pro-PGB expression, also controls the expression of the native extracellular protease AprE. AprE is produced and secreted during the early stationary phase [34], similar to pro-PGB. The highest PG activity was found in the 007 strain, which was also stable until the end of cultivation (120 h). *B. subtilis* isolates can inherit different peptidase variants with distinct activity [34] and since *B. subtilis* 007 showed high proteolytic activity [25], this might have caused an increased pro-PGB hydrolysis. At the same time, a certain stability of PGB against proteolytic degradation was shown, as PG activity was stable during the stationary phase for more than 24 h (Fig. 1b). A delayed appearance of extracellular PG activity as well as an overall low PG activity was determined in *B. subtilis* RIK1285, probably caused by the absence of AprE and NprE. Kawamura et al*.* [35] reported that this kind of *B. subtilis* double mutant only shows 4% of the native proteolytic activity. Additionally, the culture supernatant of RIK1285 contains a lot of different proteins and a clear PGB or pro-PGB band did not appear. The highest PGB activity was determined with *B. subtilis* 007 as the expression host using AUG as a start codon, therefore, this variant was used for further experiments.

### Foam Control During the Cultivation of *B. subtilis* 007 for PGB Production

A major challenge in *B. subtilis* fermentation is the high oxygen demand and the associated excessive foaming. The latter is strongly connected to the production of the surface-active compound surfactin [36, 37]. Surfactin molecules accumulate at gas–liquid interfaces, form nanostructures which stabilize the introduced air as bubbles, which ultimately form a foam [38]. At the same time, the surface tension of the culture medium is lowered [39], the oxygen introduced cannot be held within the liquid phase [40] and the oxygen demand increases. The expression of surfactin is based on several genes located in the *srfA* operon and the *sfp* locus [41]. *B. subtilis* 168 and its descendant RIK1285 inherit a non-functioning *sfp* pseudogene. The *sfp* gene is one of the essential genes for surfactin production [37], thus, foaming is not an issue for most of these research strains. In this work, however, the isolated *B. subtilis* strain 007 was used for PGB production and had already been reported to show immoderate foam formation [25]. This might indicate a functional *sfp* gene in the genome of this strain. Since *B. subtilis* 007 was found to be the best PGB producer, its excessive foaming needed to be handled short-term to ensure a stable cultivation process. An alternative approach was described by Zhang et al*.* [38], who showed that a knock-out of *srfA*-C led to a decrease in the necessity of utilizing an anti-foaming agent to only 12.2% of the amount needed initially. The knock-out of *srfA-C* might be a possibility in the future to improve the *B. subtilis* 007 strain regarding fermentation applicability.

### Anti-foaming Agent 204 for Foam Control in *B. subtilis* 007 Cultivation for Secretory PGB Production

Anti-foaming agents are used in fermentation for foam control. Anti-foam 204 (Merck, USA) is a widely used agent and, according to the manufacturer, is a polypropylene-based dispersion of polyethers that are used as a tenside to suppress foaming in fermenters. A starting concentration of 0.005–0.01% (v/v) is recommended by the manufacturer. The influence of different concentrations of the anti-foaming agent 204 (up to 0.7% (v/v)) on the cell growth of *B. subtilis* 007 and PG activity in the supernatant was investigated in a preliminary shake flask cultivation (Fig. S3). A similar growth behavior was observed compared to the control cultivation without added anti-foam and a maximal OD_600_ of 31–35 was reached within 92 h. Furthermore, the addition of anti-foaming agent 204 did not have a negative effect on the PG activity and, thus, it was found to be a suitable anti-foaming agent for bioreactor cultivations of the recombinant *B. subtilis* 007.

**Fig. 2:**
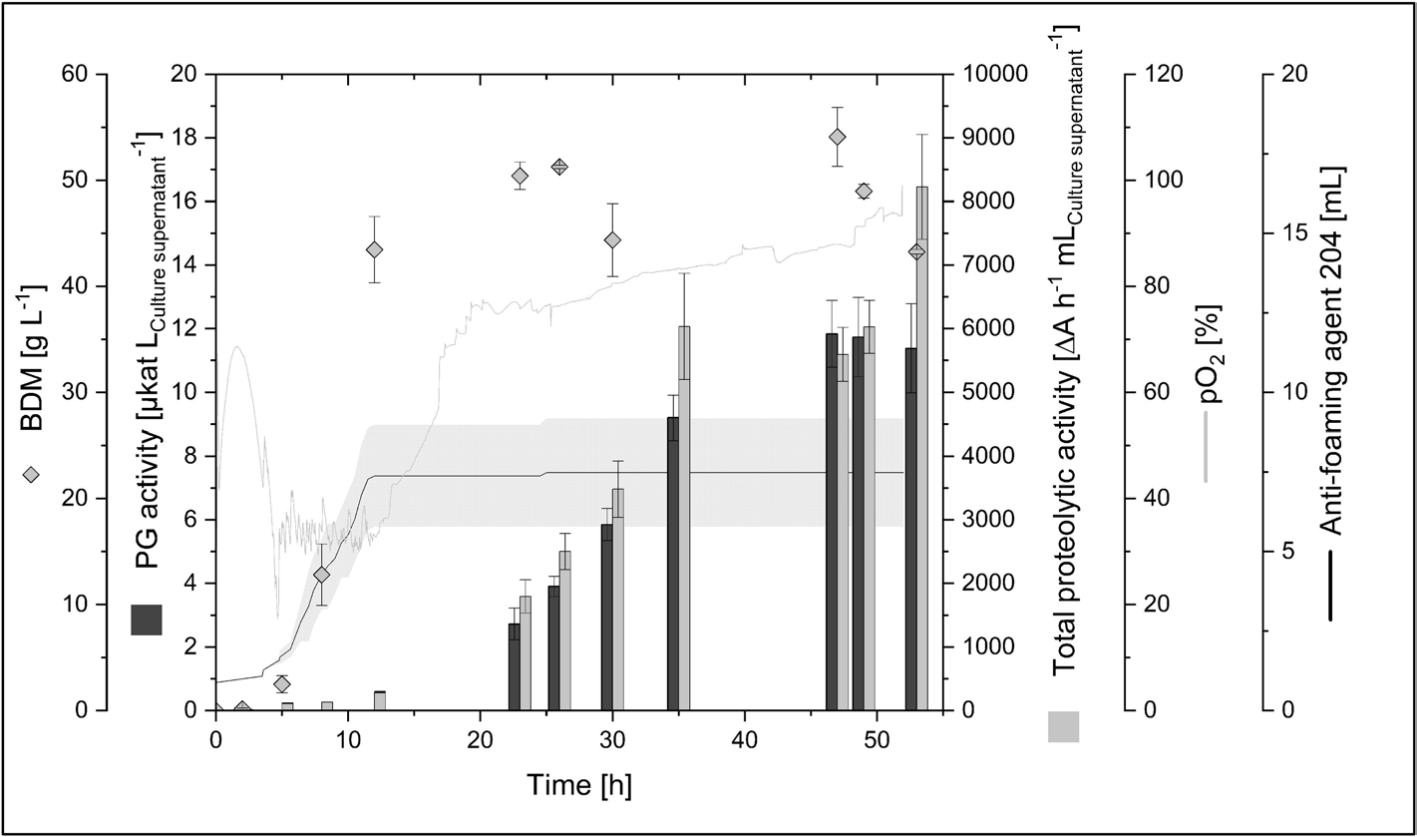
Bioreactor cultivation of recombinant *B. subtilis* 007 for secretory PGB production. Cultivation was done in duplicate in 1 L Multifors bioreactors (working volume: 0.8 L) at 30 °C and pH 6.9 ± 0.1. Total proteolytic activity was determined using azocasein as a substrate. The anti-foaming agent 204 was added manually. Deviation between the two reactors is shown in grey

Accordingly, the recombinant *B. subtilis* 007 expressing pro-PGB was cultivated in a bioreactor using anti-foam 204 for foam control (Fig. 2, Table 3). The exponential growth phase started 5 h after inoculation and transitioned into the stationary phase after 12 h. A µ_max_ of 0.3 h^−1^ was reached during exponential growth, corresponding to a generation time g of 132 min. The cell mass increased continuously until the stationary phase was reached, resulting in a bio dry mass of 52 ± 4.0 g L^−1^. It was necessary to add the anti-foaming agent 204 excessively during the exponential growth. Additionally, it was necessary to increase the stirring speed continuously and to the maximal possible extent to keep the pO_2_ above 30%. Finally, 7.4 ± 1.7 mL of anti-foaming agent 204 were added to the two reactors.

**Table 3:** Comparison of the bioreactor cultivations of *B. subtilis* 007 for secretory PGB production depending on the anti-foaming agent used (anti-foaming agent 204 and rapeseed oil). Cultivations were each done in two Multifors reactors (Working volume: 0.8 L at 30 °C)

		**Anti-foam 204**	**Rapeseed oil**
Starting conc anti-foaming agent	[% (v/v)]	0.01	0.25
Final conc anti-foaming agent	[% (v/v)]	~1–2	~ 7–8
Max. bio dry mass	[g L^−1^]	52 ± 4.0	36 ± 2
maximal growth rate (*µ*_max_)	[h^−1^]	0.30	0.33
Generation time (g)	[min]	132	124
Max. PG activity	[*µ*kat L_Culture supernatant_^−1^]	11.8 ± 1.1	7.6 ± 0.2

The PGB activity was first discovered at the end of the exponential growth phase (23 h), while the activity of the endogenous peptidases (total proteolytic activity) was first detected after 12 h (Fig. 2). The SDS-PAGE analysis indicated that pro-PGB was transported out of the cell, designated by a prominent protein band of ~ 33 kDa after 12 h of cultivation (Fig. S4). The band at ~ 33 kDa disappeared after 23 h of cultivation, while a band at ~ 21 kDa showed up, which might correspond to PGB (21 kDa). This protein band was analyzed by nano liquid chromatography tandem mass spectrometry and most of the peptides obtained verified the presence of the mature PGB (PGB amino acid sequence coverage: 85%; Fig. S5). One peptide found in this analysis originated from the propeptide and might be an artefact or a peptide which was bound to the PGB even after cleavage. PGB activity leveled off during the stationary phase and its maximum was reached after 47 h with 11.8 ± 1.1 µkat L_Culture supernatant_^−1^ (Fig. 2). The transcription of the pro-PGB gene was facilitated by the same promoter (P_aprE_), as *B. subtilis*’ most abundant extracellular peptidase AprE. This P_aprE_ promoter is a constitutive promoter which is maximally active in the early stationary phase [42]. The total proteolytic activity increased strongly with the onset of the stationary phase. The pro-PGB expression most probably also started at the beginning of the stationary phase. However, the pro-PGB shows a ~17-fold lower PG activity in comparison to the mature PGB [14] and, thus, deamidating activity was detectable after some delay, as the propeptide needed to be cleaved by the *B. subtilis* peptidases.

#### Rapeseed Oil for Foam Control in *B. subtilis* 007 Cultivation for Secretory PGB Production

Using plant oil as an anti-foaming agent can be a cost-effective and more sustainable alternative to conventional anti-foaming agents. Therefore, rapeseed oil was tested for pro-PGB production in *B. subtilis* 007, and the activity yield was compared to the cultivation described previously when anti-foaming agent 204 was used (Table 3).

**Fig. 3:**
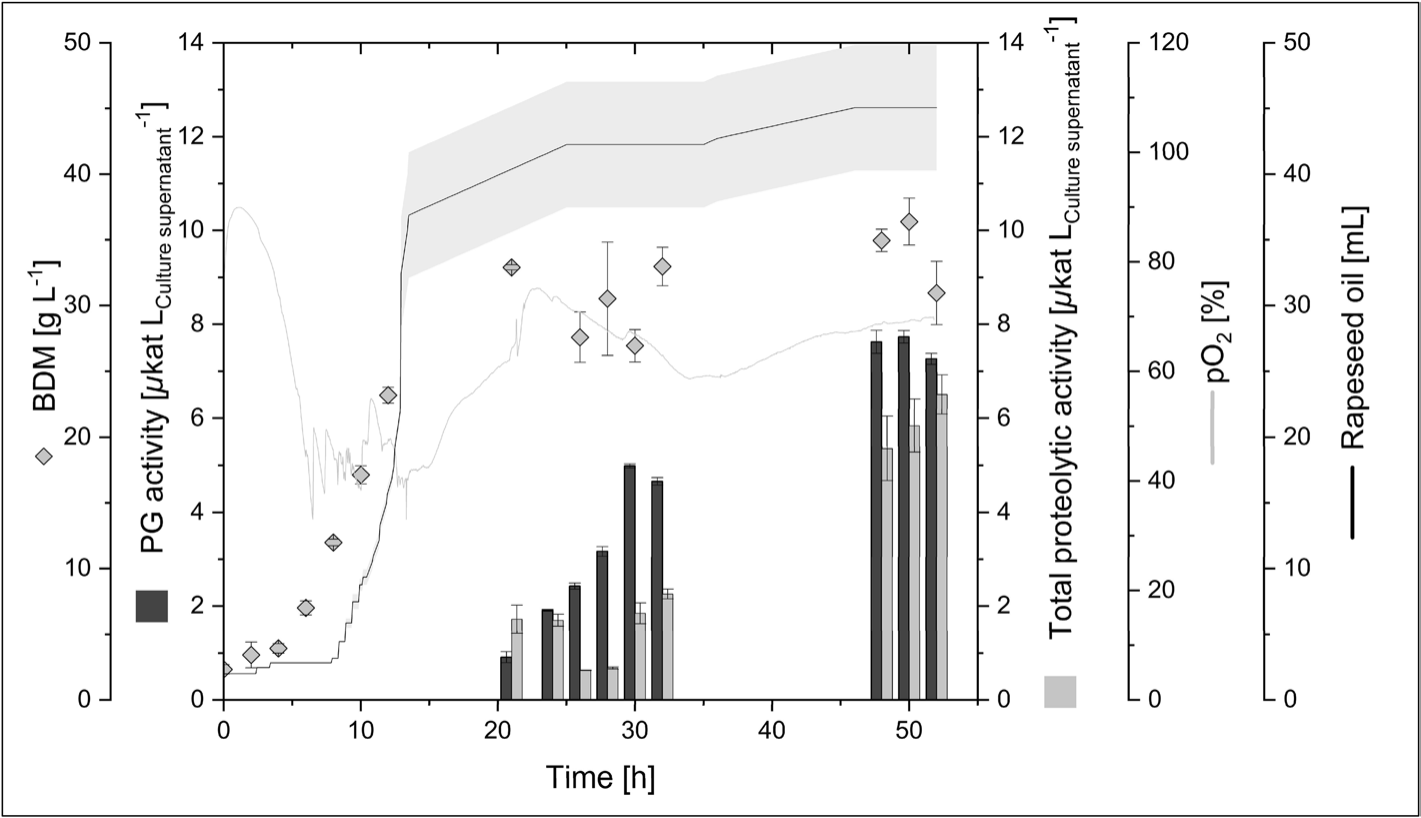
Bioreactor cultivation of recombinant *B. subtilis* 007 for secretory PGB production. Cultivation was performed in 1 L Multifors bioreactors (working volume: 0.8 L) at 30 °C and pH 6.9 ± 0.1. Total proteolytic activity was determined using Suc-Ala-Ala-Pro-Phe-pNA as a substrate. Rapeseed oil was added manually when needed and fed from 11 to 25 h and 36 to 47 h. Deviation between the two reactors is shown in grey

A concentration of 0.25% (v/v) rapeseed oil had already been added to the cultivation medium before inoculation and was increased during cultivation when required. An amount of 9.4 ± 0.6 mL of rapeseed oil was given to the reactors until 11 h after inoculation. By using a consistent feed of rapeseed oil applied from 11 to 25 h (10 s every 5 min at a flow rate of 47 µL s^−1^) and from 36 to 47 h (10 s every 30 min at a flow rate of 47 µL s^−1^), a total volume of 45 ± 4.8 mL of rapeseed oil was added until the cultivation was stopped after 52 h. This is up to eightfold higher than the volume of anti-foaming agent 204 used. Similar to the cultivation with anti-foaming agent 204, the exponential growth phase started after 5 h and ended after 21 h (Fig. 3). A lower bio dry mass was reached when using rapeseed oil, while the µ_max_ remained similar (0.3 and 0.33 h^−1^) for both cultivations (Table 3). The maximal volumetric PGB activity was lower compared to the cultivation conducted with anti-foaming agent 204. As seen previously, PGB activity and total proteolytic activity was determined in the supernatant at the end of exponential growth. The SDS-PAGE analysis (Fig. S6) showed a secretion of pro-PGB (33 kDa) and subsequent propeptide cleavage by *Bacillus’* peptidases to form mature PGB (21 kDa).

Several research groups investigated different *B. subtilis* strains for production of PG variants. In these studies, PG activity is expressed in “enzyme units,” whereas in the present study, the SI unit katal is used [43]. To ensure better comparability, the PG activities reported in this study were converted into enzyme units (Table 4).

**Table 4:** Summary of studies focusing on PG production using *B. subtilis* as expression host. *pro-PG processed to active PG using trypsin

Origin of protein-glutaminase	Produced PG variant	Production host	Production scale	Activity yield [U/mL_Culture supernatant_^−1^]	Reference
*C. proteolyticum*	Pro-PG*	*B. subtilis* WB600	Shake flask	0.2	[46]
	Pro-PG-His_6_	*B. subtilis* WB600	Shake flask	1.2	[24]
			Bioreactor (5 L)	7.1	
	sfGFP-pro-PG*	*B. subtilis* WB800N	unknown	37	[44]
	Pro-PG-His_6_	*B. subtilis* 168	unknown	0.6	[18]
	Pro-PG-His_6_ (A291S)	*B. subtilis* 168 Δdal	Shake flask	5.3	[45]
			Bioreactor (5 L)	8.2	
*B. helcogenes*	Pro-PG	*B. subtilis* 007	Bioreactor (0.8 L)	0.7	This study

Usually, low extracellular proteolytic activity is optimal for the production of recombinant proteins, with the aim of preventing the degradation of the target protein. Consequently, *Bacillus* strains exhibiting diminished extracellular proteolytic activity, including *B. subtilis* WB600 and WB800, were examined for their ability to produce PG. A maximal PG activity of 7.1 U mL^-1^ was obtained in *B. subtilis* WB600 in a 5-L bioreactor [24]. The so far highest PG activity yield of 37 U mL^-1^ was achieved in *B. subtilis* WB800N after extensive optimization of the promoter and ribosomal binding site [44]. However, the usage of such strains requires the additional step of propeptide hydrolysis to obtain catalytically active PG. In strains exhibiting extracellular proteolytic activity, such as *B. subtilis* 168, maximal PG activity yields of 0.6 U mL^-1^ have been observed [18]. A mutant variant (A291S) of *C. proteolyticum* PG showed an enhanced PG activity yield of 8.2 U/mL when produced in a food grade *B. subtilis* strain [45]. The authors hypothesized an altered substrate entry into the catalytic site for this mutant variant. For PG from *B. helcogenes*, a PG activity yield of 0.7 U mL^-1^ was observed to be comparable to the PG activity yield of *C. proteolyticum* PG in unmodified *B. subtilis* 168. However, *B. subtilis* 007 as well as the PGB expression cassette itself were unmodified and not optimized so far.

### Partial PGB Purification via Hydrophobic Interaction Chromatography

The PGB from shake flask cultivation supernatant was first concentrated by fractionated ammonium sulphate precipitation, as described by Horstmann et al*.* [13], and further purified by HIC. The specific PG activity in this first step of crude protein precipitation was increased from 6.7 to 22 nkat mg^−1^ with a yield of 57% (Table 5). A similar yield of 61% was achieved for pro-PGB produced in *E. coli* and concentrated similarly by fractionated ammonium sulphate precipitation [13]. The specific PG activity was increased by HIC purification to 28.6 nkat mg^−1^ (purification factor of 4.3-fold) with a final yield of 14.8% (for chromatogram, see Fig. S7).

**Table 5:** Purification of PGB via hydrophobic interaction chromatography

Step	Spec. PG activity [nkat mg^−1^]	Purification factor [-]	Yield [%]
Culture supernatant	6.7	1.0	100.0
Ammonium sulfate precipitation	21.5	3.2	57.1
PG-containing peak	28.6	4.3	14.8

**Fig. 4:**
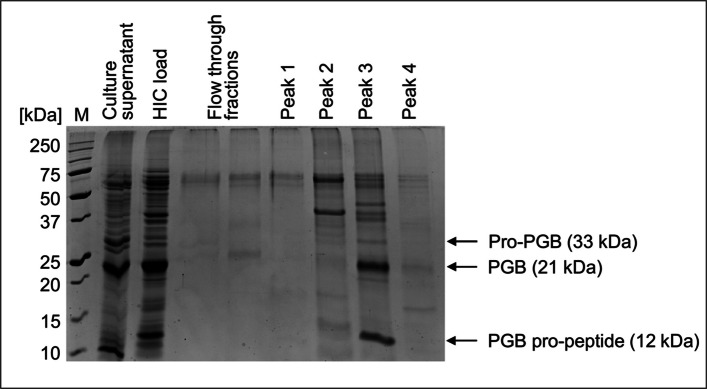
Sodium dodecyl sulfate polyacrylamide gel electrophoresis (SDS-PAGE) analysis (12.5% acrylamide gel, protein load: 5 µg) of the PGB purification from the shake flask cultivation supernatant of recombinant *B. subtilis* 007. HIC load: Fractionated ammonium sulphate precipitation of cultivation supernatant. Flow-through fractions and peaks 1–4 refer to the subsequent HIC purification. M: Protein marker

The SDS-PAGE analysis revealed that the final sample (‘peak 3’) contained two prominent protein bands (Fig. 4), apart from some high-molecular nontarget proteins. The two intensive bands most probably refer to the mature PGB at 21 kDa as well as the supposed-to-be cleaved propeptide at 12 kDa. This suggests that the propeptide is either concentrated along with the mature PGB or still attached to the mature PGB even though it had been cleaved off. Lu et al*.* [17] observed the same phenomenon with PGC produced with a 6xHistidine- and maltose-binding-protein-tag. Here, a washing step with 4 M urea was necessary to remove the propeptide from the mature PGC.

The purification described here was not sufficient to purify PGB to homogeneity. However, if this was needed for further experiments, the HIC purification method described here might be useful as a starting point for further development. Regarding a more industrially relevant process, however, the PGB was also purified by cross-flow filtration.

### *Partial Purification of PGB *via* Cross-flow Filtration*

The bioreactor cultivation supernatant containing anti-foaming agent 204 was partially purified by cross-flow filtration. Firstly, the supernatant was concentrated (2.3-fold) with a 30 kDa MWCO cross-flow membrane, resulting in a PG activity yield of 84%. Only 0.8% of PG activity was found in the 30 kDa filtrate. The specific PG activity did not increase during filtration, as this process did not remove any of the nontarget proteins. Additionally, the secreted peptidases of *B. subtilis* were concentrated alongside the PGB, leaving 83% of the initial total proteolytic activity after cross-flow filtration. The concentrated supernatant was heated to 60 °C for 1 h to inactivate the *Bacillus*’ peptidases. This step lowered the total proteolytic activity to < 5% compared to the initial activity. The heat treatment resulted in a loss of 28% of PGB activity, while the specific PGB activity increased from 2.6 to 3.9 nkat mg^−1^ due to the precipitation of heat-labile proteins. A previous study showed that pro-PGB seems to be distinctively more stable, as 96% of PG activity was retained after incubation at 60 °C for 4 h [13]. However, it must be considered that pro-PGB was not produced in *B. subtilis*, but in *E. coli* instead. The PGB seems to be relatively stable towards proteolytic degradation, as can be seen in the bioreactor cultivation. However, the influence of the residual proteolytic activity needs to be investigated for the long-time storage of the concentrated PGB.

**Fig. 5:**
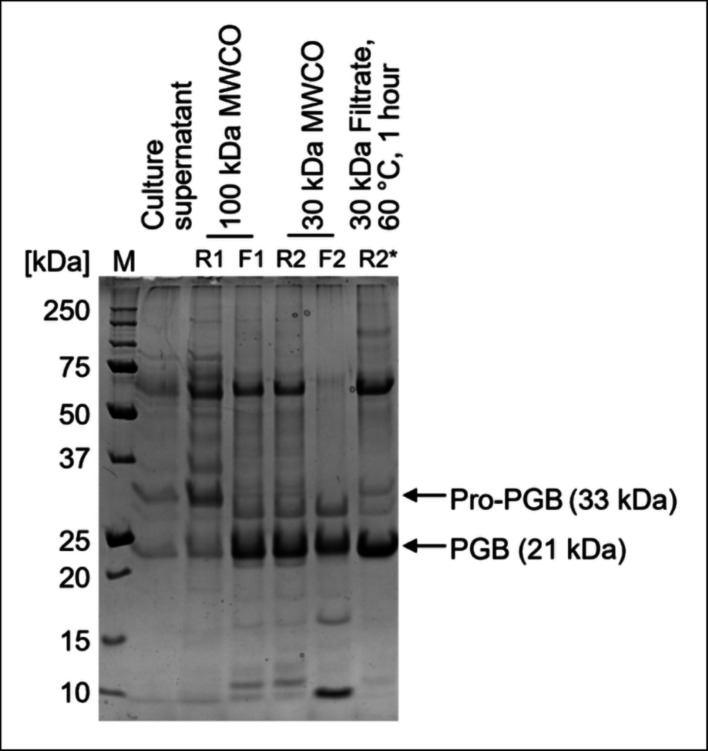
SDS-PAGE analysis (12.5% acrylamide gel, protein load: 5 µg) of partial PGB purification from *B. subtilis* 007 bioreactor cultivation supernatant containing rapeseed oil by cross-flow filtration. Retentate 1 (R1) and filtrate 1 (F1) were obtained by filtration with a 100 kDa MWCO membrane. Retentate 2 (R2) and filtrate 2 (F2) were obtained by filtration of F1 with a 30 kDa MWCO. R2* was obtained by heating R2 to 60 °C for 1 h. M: Protein marker

The procedure had to be adjusted to process the cultivation supernatant containing rapeseed oil via cross-flow filtration. The combination of oil and excessive stirring led to an emulsion-like turbid liquid. A filtration step using a 100 kDa MWCO membrane was performed to remove the turbidities, followed by the 30 kDa filtration. A clear liquid was obtained after this procedure. The SDS-PAGE analysis (Fig. 5) revealed that a protein at the molecular weight of pro-PGB and some other proteins in the range of 33–75 kDa were retained in the retentate (R1). Pro-PGB tends to form dimers and, thus, could not cross the membrane [13]. The PGB was found to be a monomer in size exclusion experiments [14] and, as expected, PGB (21 kDa) crossed the 100 kDa MWCO membrane and was mostly detected in the filtrate with 14 nkat mL^−1^ (F1). The PGB was retained during the 30 kDa MWCO (R2) filtration and purified 3.9-fold to a specific PG activity of 15 nkat mg^−1^. Heating the 30 kDa retentate decreased the amount of nontarget proteins (R2*). Here, the heating step had a greater impact on the sample’s purity (eightfold), and it increased the specific PG activity to 32 nkat mg^−1^, while the peptidase activity was decreased to 4%. The prominent protein band of around 60 kDa, which was enriched alongside the PGB, might have crossed the membrane due to a favorable conformation (R2). A protein band of this size is also visible in immobilized metal affinity chromatography fractions of purified pro-PGB produced in *E. coli* [13] and, thus, it might be a stable multimer of PGB. The specific PG activity of 32 nkat mg^−1^ is in a similar range as that seen in previous experiments, where pro-PGB, produced in *E. coli* was hydrolyzed with trypsin and mature PGB was obtained [14].

### Deamidation of Wheat Gluten Using Concentrated PGB

**Fig. 6:**
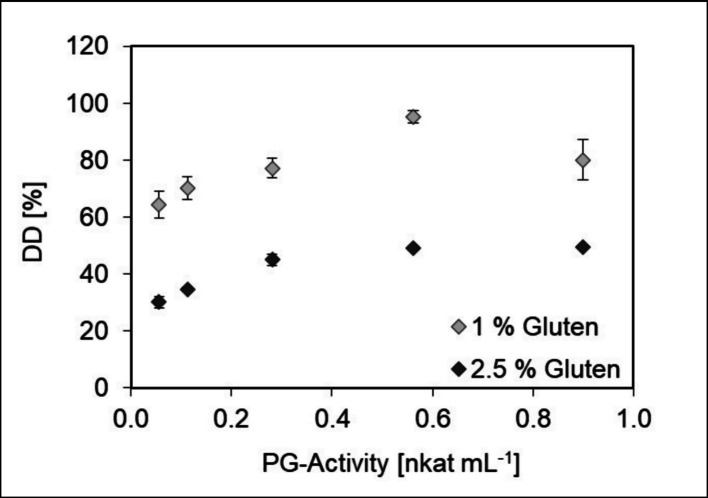
Deamidation of 1 and 2.5% (w/v) gluten suspensions for 3 h using increasing PG activities. Deamidation was conducted at 50 °C and stopped by incubation at 95 °C for 5 min. DD [%] = degree of deamidation

The deamidation of a protein can lead to an improvement of its solubility and, as a result, alter its technofunctional properties [9, 11]. Wheat gluten contains a high number of glutamine residues and was, therefore, used as a substrate for deamidation experiments using PGB (partially purified via cross-flow filtration). Gluten suspensions of 2.5 and 1% (w/v) were incubated with PGB activities of 0.06–0.9 nkat mL^−1^ (3 h, 50 °C). A maximal deamidation degree (DD%) of 49 ± 2% was reached for the 2.5% (w/v) gluten suspension using PGB with 0.56 nkat mL^−1^. A maximal DD% of 95 ± 2% was obtained using the same PGB activity but decreasing the gluten concentration to 1% (w/v) (Fig. 6). Pro-PGB produced in *E. coli* BL21(DE3) and a commercially available preparation of PG from *C. proteolyticum* (PGA) were used in a previous study [13] for gluten deamidation experiments. For deamidation of a 5% (w/v) gluten suspension, both PG’s reached similar DD% of 46% (pro-PGB) and 44% (PGA), respectively. In a 2.5% (w/v) gluten suspension, DD% of 96% (pro-PGB) and 83% (PGA) were reached in 24 h. Pro-PGB deamidated gluten faster, reaching a plateau in DD% after five hours. For PGA, the plateau was probably reached between eight and 25 h of deamidation.

The former experiments [13] showed that the maximal possible DD% decreases with increasing substrate concentration in case of gluten. This was explained by the total amount of ammonia released during the deamidation reaction, which inhibited the deamidation activity of pro-PGB. The deamidation experiments described in this study showed that PGB was inhibited by lower ammonia concentrations compared to pro-PGB. While pro-PGB tolerated gluten concentrations of up to 2.5% without recognizable inhibitory effects, PGB was already inhibited at this concentration. This might also be the reason for the slower deamidation of PGA described in [13], as the preparation contains mature PGC.

## Conclusion

Three *B. subtilis* strains were investigated for recombinant PGB production and simultaneous propeptide hydrolysis, where the isolated *B. subtilis* 007 was found to be the most suitable strain. Two anti-foaming agents were tested to control this strain’s excessive foaming. Sufficient activity was obtained using the commercial antifoam agent 204 (11.8 ± 1.1 µkat L_Culture supernatant_^−1^) and rapeseed oil (7.6 ± 0.7 µkat L_Culture supernatant_^−1^), respectively. Rapeseed oil, as the less cost-intensive alternative, did not alter the µ_max_ in the bioreactor cultivation and a good activity yield was still achieved. *B. subtilis* 007 can be genetically modified for further experiments to prevent excessive foaming in bioreactors. Two ways of downstream processing were tested: protein precipitation followed by HIC and cross-flow filtration. A partially purified PGB preparation was obtained and gluten deamidation experiments were performed. This illustrated PGB’s relevance for the food industry as mature PGB can be applied to deamidate gluten to a similar %DD as pro-PGB in less time.

## Supplementary Information

Below is the link to the electronic supplementary material.

Supplementary file1 (DOCX 2482 KB)

## Data Availability

The data is available in the main manuscript and in the supplementary file if mentioned.
