# Supplementary material for: Secretory Production of Mature Protein-Glutaminase from Bacteroides helcogenes in Bacillus subtilis
Source: Appl Biochem Biotechnol. 2026 Mar 2;198(5):3747–67. doi: 10.1007/s12010-026-05637-6 (PMC13139226; doi:10.1007/s12010-026-05637-6)
Supplement: Supplementary file 1 — Supplementary file1 (DOCX 2482 KB) [file 12010_2026_5637_MOESM1_ESM.docx]

**Secretory production of mature protein-glutaminase from
*Bacteroides helcogenes* in *Bacillus subtilis***

**Gudrun Horstmann, Nicole Roth, Eva Pross, Jana Senger, Ines Seitl, Lucas Kettner, Lutz Fischer^#^**

University of Hohenheim, Institute of Food Science and Biotechnology,
Department of Biotechnology and Enzyme Science, Garbenstrasse 25, 70599 Stuttgart, Germany

^#^ Corresponding author: Tel.: +49 711 459 23018; E-mail address: sekretariat-bt@uni-hohenheim.de

**Supplements**

**Table S1** Primers used in this study

| PCR template | Primer | Sequence^a^ |
| --- | --- | --- |
| Pro-PGB gene in gDNA of *B. helcogenes* | Pro-PGB_*BssH*II_fw | 5’ GT GCG CGC TGC ACA CAC GAT GAT AAT AAT G 3’ |
|  | PGB_*Xho*I_rv | 5’ G ATG CTC GAG TTA AAA TCC ACA TGA AAC TGT A 3’ |
| pLF vector containing P_aprE_ and PhoD signal sequences | QC_M1V_fw | 5’ GGG TAA CTA ATG GCA TAC GAC 3’ |
|  | QC_M1V_rv | 5’ GTC GTA TGC CAT TAG TTA CCC 3’ |

^a^ recognition sites are underlined

**
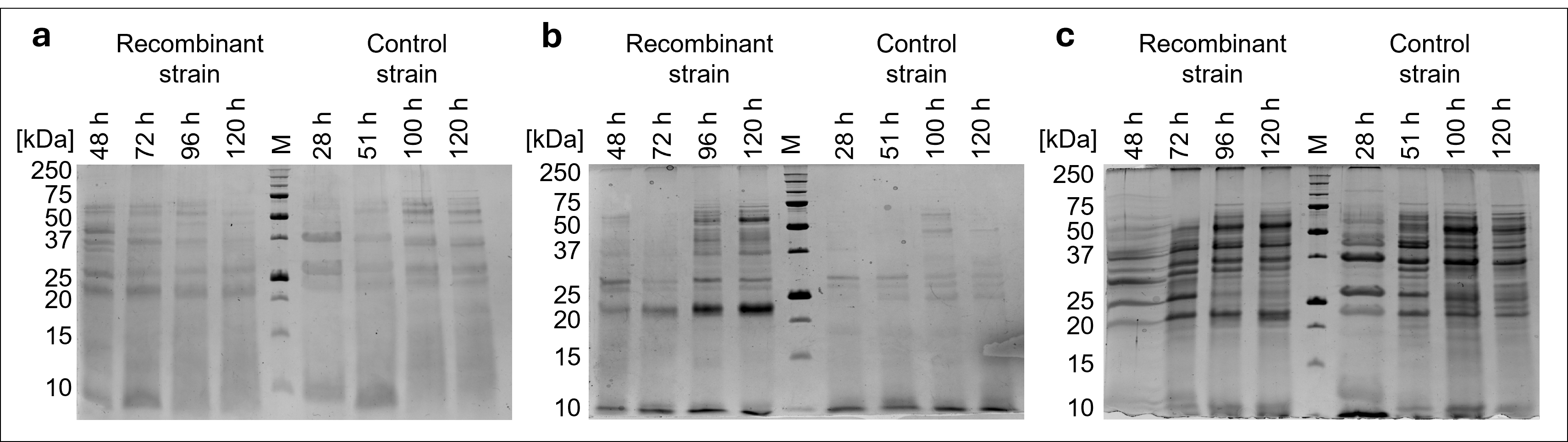
Fig. S1** Cultivation progress of recombinant *B. subtilis* 168 (a), 007 (b) and RIK1285 (c) producing pro-PGB (expression plasmid: pLF_AUG_pro-PGB, start codon: AUG) and respective SDS-PAGE analysis (12.5 % acrylamide gel, protein load: 5 µg) of cultivation supernatant. M: Protein marker. Theoretical molecular weights: Pro-PGB: 33 kDa; PGB: 21 kDa; Pro-peptide: 12 kDa

**
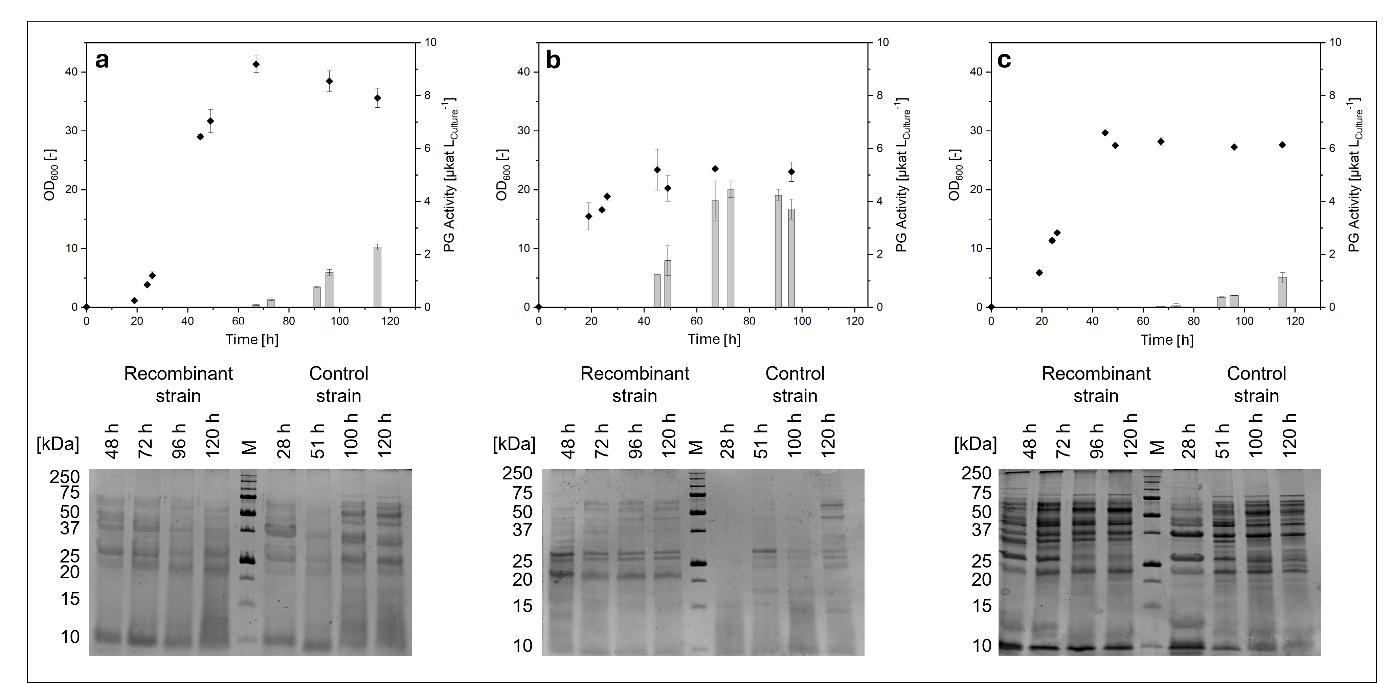
**

**Fig. S2** Cultivation progress of recombinant *B. subtilis* 168 (a), 007 (b) and RIK1285 (c) producing pro-PGB (expression plasmid: pLF_GUG_pro-PGB, start codon: GUG) and respective SDS-PAGE analysis (12.5 % acrylamide gel, protein load: 5 µg) of cultivation supernatant. M: Protein marker. Theoretical molecular weights: Pro-PGB: 33 kDa; PGB: 21 kDa; Pro-peptide: 12 kDa

**
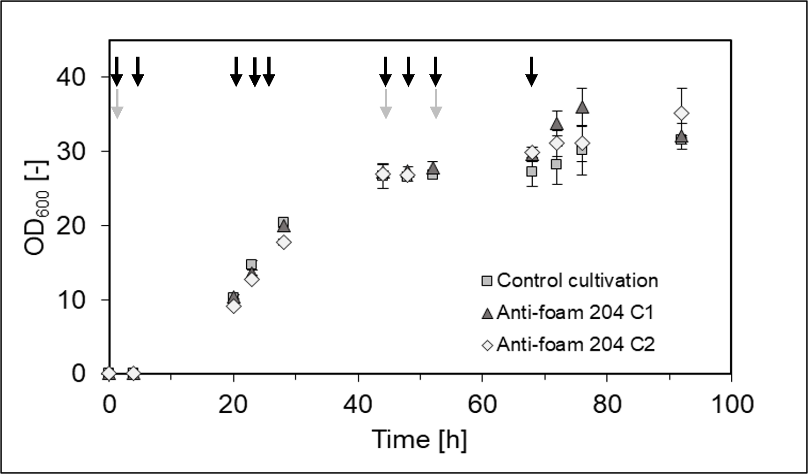
**

**Fig. S3** Influence of anti-foaming agent 204 on the growth of recombinant *B. subtilis* 007 for PGB production. Cultivation was done in duplicate shake flasks. Black and grey arrows: anti-foaming agent 204 addition in cultivation 1 (C1) and 2 (C2), respectively


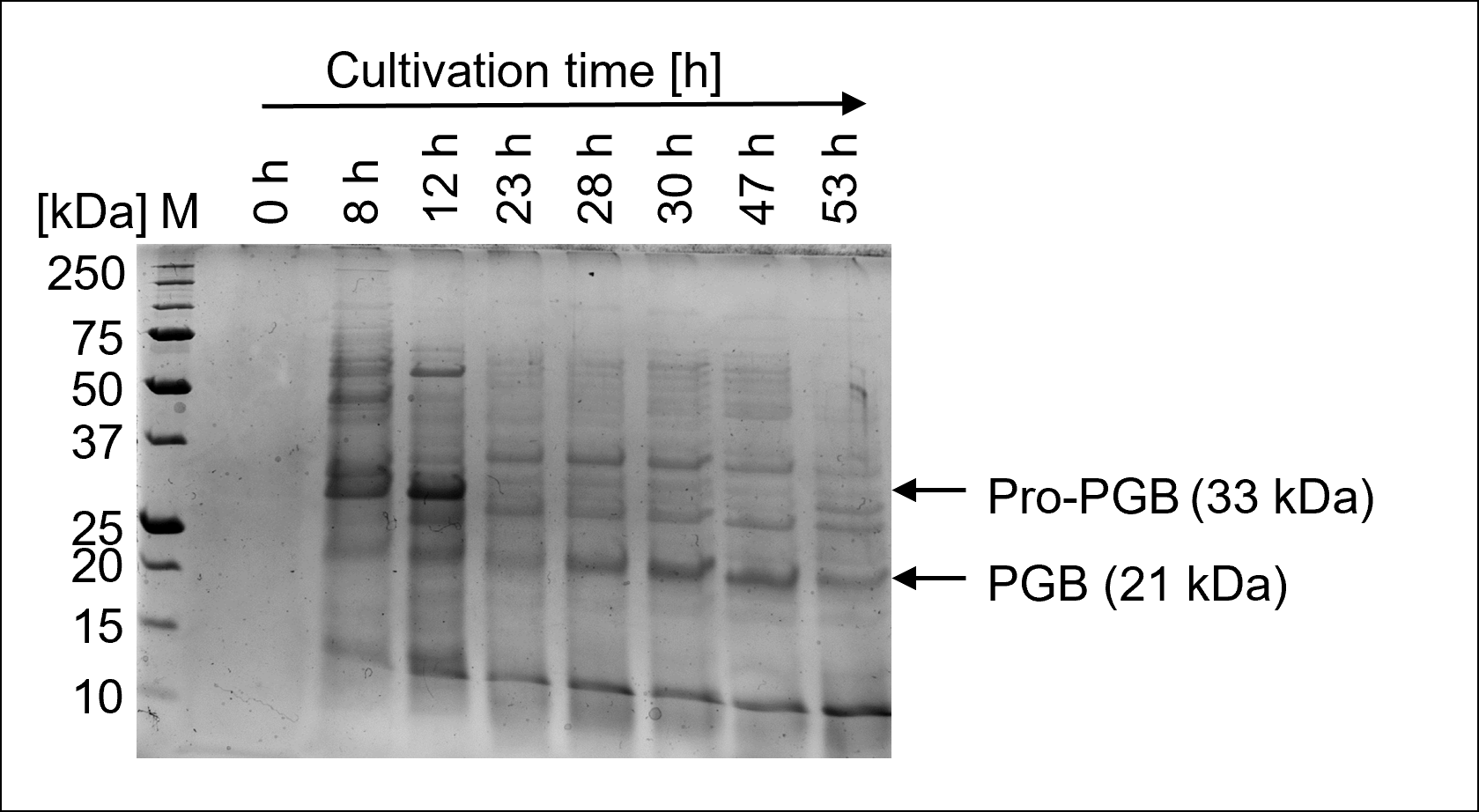


**Fig. S4** SDS-PAGE analysis (12.5 % acrylamide gel, protein load: 5 µg) of supernatants from the bioreactor cultivation of PGB-producing *B. subtilis* 007. Anti-foaming agent 204 was used for foam control. M: Protein marker. Theoretical molecular weights: Pro-PGB: 33 kDa; PGB: 21 kDa

**
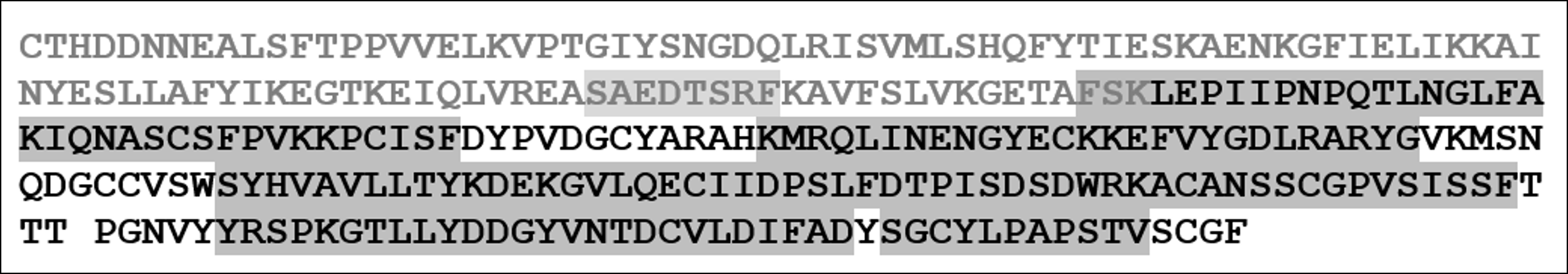
**

**Fig. S5** Peptides found in the MS analysis of the PGB protein band. Grey background shows the entirety of the peptides found in relation to the pro-PGB amino acid sequence. Grey letters indicate the PGB pro-peptide sequence and black letters indicate the sequence of mature PGB

**
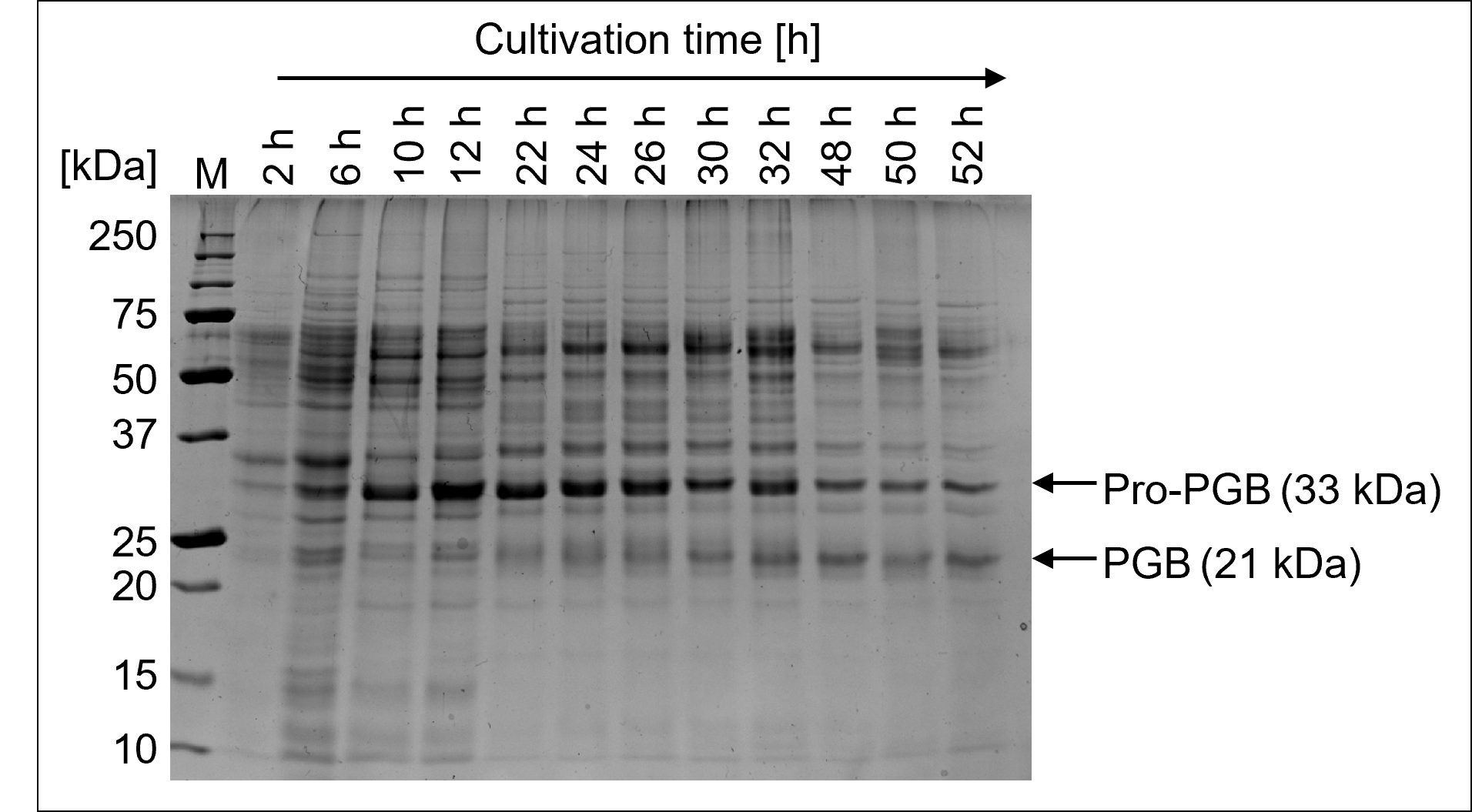
**

**Fig. S6** SDS-PAGE analysis (12.5 % acrylamide gel, protein load: 5 µg) of supernatants from the bioreactor cultivation of PGB-producing *B. subtilis* 007. Rapeseed oil was used for foam control. M: Protein marker. Theoretical molecular weights: Pro-PGB: 33 kDa; PGB: 21 kDa

**
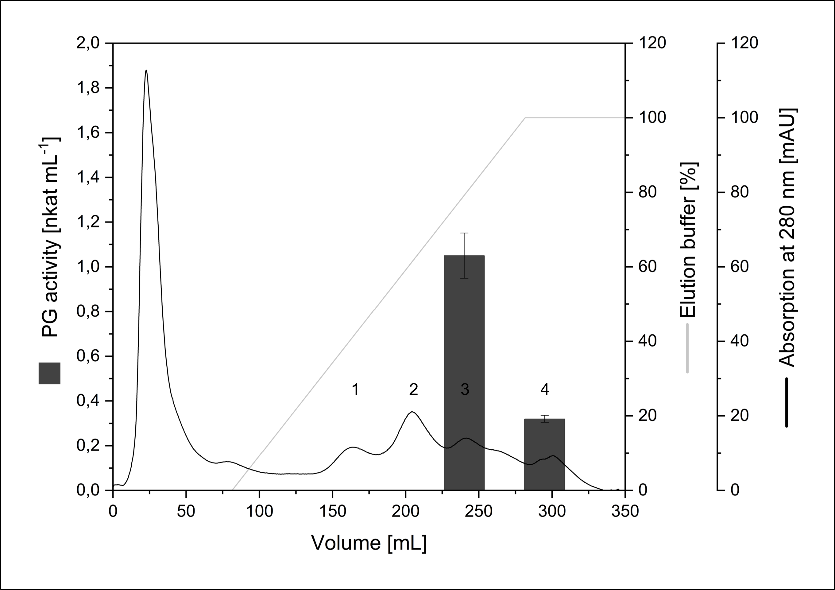
**

**Fig. S7** Chromatogram of the PGB purification by hydrophobic interaction chromatography. Elution buffer: 25 m*M* sodium phosphate buffer (pH 7). Relevant peaks are labeled 1 – 4 and were investigated for PG activity
